# Refined Chelator Spacer Moieties Ameliorate the Pharmacokinetics of PSMA-617

**DOI:** 10.3389/fchem.2022.898692

**Published:** 2022-08-09

**Authors:** José Carlos dos Santos, Martin Schäfer, Ulrike Bauder-Wüst, Barbro Beijer, Matthias Eder, Karin Leotta, Christian Kleist, Jan-Philip Meyer, Thomas R. Dilling, Jason S. Lewis, Clemens Kratochwil, Klaus Kopka, Uwe Haberkorn, Walter Mier

**Affiliations:** ^1^ Department of Nuclear Medicine, Heidelberg University Hospital, Heidelberg, Germany; ^2^ Research Group Molecular Biology of Systemic Radiotherapy, German Cancer Research Center, Heidelberg, Germany; ^3^ Department of Nuclear Medicine, Division of Radiopharmaceutical Development, University Medical Center, University of Freiburg, Freiburg, Germany; ^4^ German Cancer Consortium, Partner Site Freiburg, University Medical Center, Freiburg, Germany and German Cancer Research Center, Heidelberg, Germany; ^5^ Clinical Cooperation Unit Nuclear Medicine, German Cancer Research Center, Heidelberg, Germany; ^6^ Department of Radiology and the Program in Pharmacology, Memorial Sloan Kettering Cancer Center, New York, NY, United States; ^7^ Institute of Radiopharmaceutical Cancer Research, Helmholtz-Zentrum Dresden-Rossendorf, Dresden, Germany

**Keywords:** PSMA, prostate cancer, PET imaging, endoradiotherapy, chelator

## Abstract

Prostate-specific membrane antigen (PSMA) binding tracers are promising agents for the targeting of prostate tumors. To further optimize the clinically established radiopharmaceutical PSMA-617, novel PSMA ligands for prostate cancer endoradiotherapy were developed. A series of PSMA binding tracers that comprise a benzyl group at the chelator moiety were obtained by solid-phase synthesis. The compounds were labeled with ^68^Ga or ^177^Lu. Competitive cell-binding assays and internalization assays were performed using the cell line C4-2, a subline of the PSMA positive cell line LNCaP (human lymph node carcinoma of the prostate). Positron emission tomography (PET) imaging and biodistribution studies were conducted in a C4-2 tumor bearing BALB/c nu/nu mouse model. All ^68^Ga-labeled ligands were stable in human serum over 2 h; ^177^Lu-CA030 was stable over 72 h. The PSMA ligands revealed inhibition potencies [K_i_] (equilibrium inhibition constants) between 4.8 and 33.8 nM. The percentage of internalization of the injected activity/10^6^ cells of ^68^Ga-CA028, ^68^Ga-CA029, and ^68^Ga-CA030 was 41.2 ± 2.7, 44.3 ± 3.9, and 53.8 ± 5.4, respectively; for the comparator ^68^Ga-PSMA-617, 15.5 ± 3.1 was determined. Small animal PET imaging of the compounds showed a high tumor-to-background contrast. Organ distribution studies revealed high specific uptake in the tumor, that is, approximately 34.4 ± 9.8% of injected dose per gram (%ID/g) at 1 h post injection for ^68^Ga-CA028. At 1 h p.i., ^68^Ga-CA028 and ^68^Ga-CA030 demonstrated lower kidney uptake than ^68^Ga-PSMA-617, but at later time points, kidney time–activity curves converge. In line with the preclinical data, first diagnostic PET imaging using ^68^Ga-CA028 and ^68^Ga-CA030 revealed high-contrast detection of bone and lymph node lesions in patients with metastatic prostate cancer. The novel PSMA ligands, in particular CA028 and CA030, are promising agents for targeting PSMA-positive tumor lesions as shown in the preclinical evaluation and in a first patient, respectively. Thus, clinical translation of ^68^Ga-CA028 and ^68^Ga/^177^Lu-CA030 for diagnostics and endoradiotherapy of prostate cancer in larger cohorts of patients is warranted.

## Introduction

The levels of the cell surface receptor prostate-specific membrane antigen (PSMA) are correlated with the stage and grade of prostate carcinoma (Silver et al., 1997; Milowsky et al., 2007; Morris et al., 2007). While increased expression of PSMA implies cancer progression (Yao and Bacich, 2006; Yao et al., 2010) and, most importantly, PSMA is significantly overexpressed on malignant prostate cancer cells as compared to benign prostatic hyperplasia and normal prostatic and other tissues and was even found to be upregulated after androgen-deprivation therapy, PSMA has been proven a valuable target for molecular imaging and treatment of metastatic castration-resistant prostate cancer (mCRPC) (Chen et al., 2011; Barrett et al., 2013; Zechmann et al., 2014; Afshar-Oromieh et al., 2015a; Afshar-Oromieh et al., 2015b; Kratochwil et al., 2016; Schmidkonz et al., 2018).

In recent years, several PSMA-targeting radiotracers such as ^123^I-MIP-1095 (Hillier et al., 2009), ^99m^Tc-MIP-1404 (Schmidkonz et al., 2018), ^68^Ga-PSMA-11 (Eder et al., 2012), ^68^Ga-PSMA I&T (Weineisen et al., 2015), ^68^Ga-PSMA-617 (Afshar-Oromieh et al., 2015a), ^18^F-DCFBC (Turkbey et al., 2017), ^18^F-DCFPyL (Chen et al., 2011), and ^18^F-PSMA-1007 (Cardinale et al., 2017) were found to provide higher detection rates in comparison to ^11^C- and ^18^F-choline (Hodolic, 2010) diagnostics. Among them, the urea-based peptidomimetic inhibitors showed ideal targeting properties (Eder et al., 2012; Benesova et al., 2015), whereas currently ^68^Ga-PSMA-11 is one of the most relevant tracers for detecting, staging, and restaging of recurrent lesions and for monitoring the course of treatment of mCRPC (Eder et al., 2012). However, the fact that *N,N′*-bis [2-hydroxy-5-(carboxyethyl)benzyl]-ethylenediamine-*N,N′*-diacetic acid (HBED-CC), the chelator within PSMA-11, does not form stable chelate complexes with the most relevant therapeutic radionuclides greatly limits its use as a theranostic agent.

**FIGURE 1:**
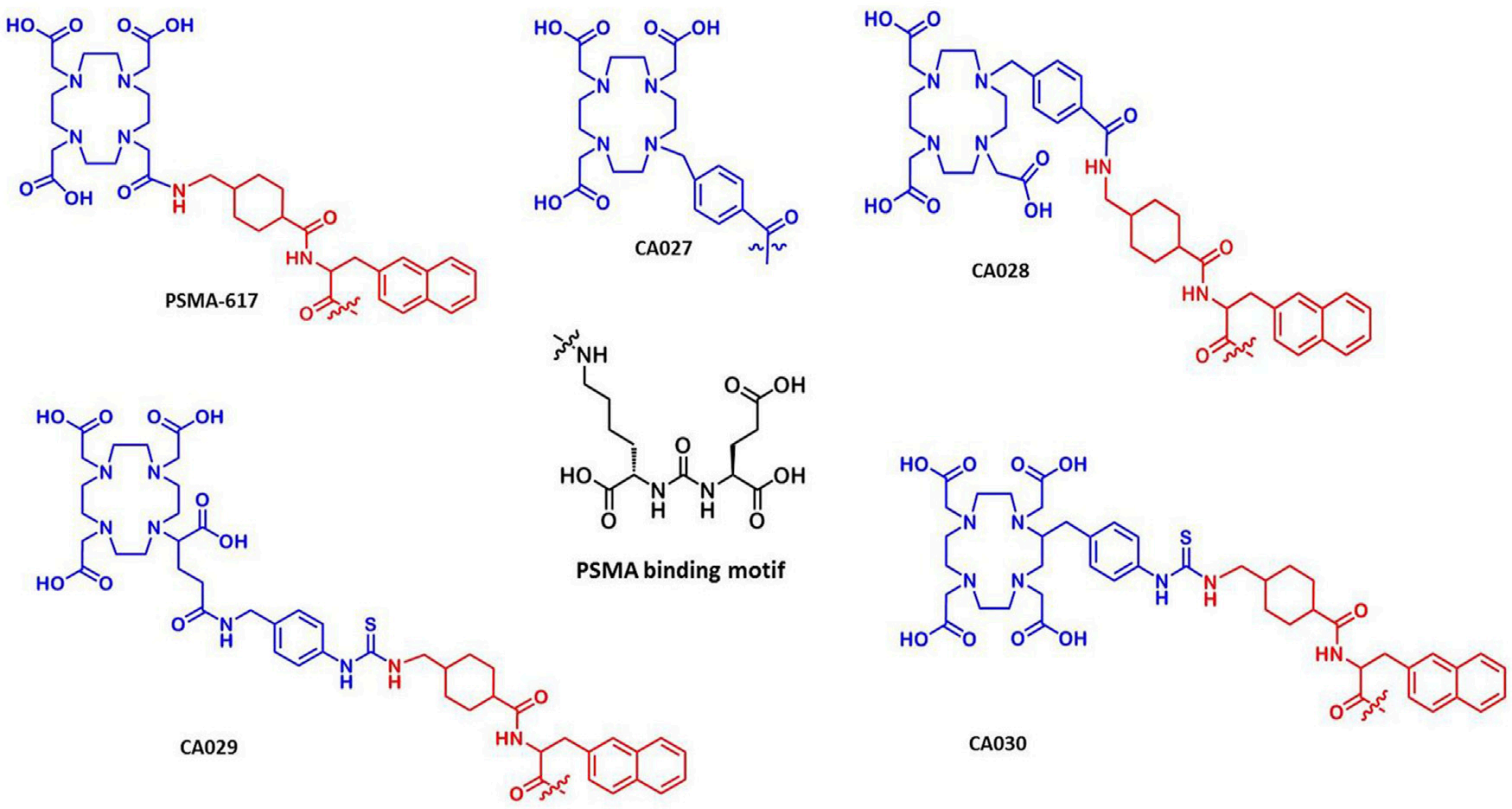
Chemical structures of the novel PSMA ligands in comparison with the reference compound PSMA-617. The ligands comprise the PSMA-specific urea-based binding motif, the linker, as compared to the reference PSMA-617.

In contrast, the compound PSMA-617 contains the chelator 1,4,7,10-tetraazacyclododecane-1,4,7,10-tetraacetic acid (DOTA), which is routinely chosen if labeling with both diagnostic (e.g., ^44^Sc, ^67/68^Ga, ^111^In) and therapeutic radionuclides (e.g., ^90^Y, ^177^Lu) is intended (Price and Orvig, 2014). Also, other ligands labeled with beta-emitters, for example, ^131^I-MIP-1095 (Zechmann et al., 2014), ^177^Lu-PSMA I&T (Weineisen et al., 2015; Yusufi et al., 2021), and ^90^Y-/^213^Bi-PSMA-617 (Benesova et al., 2015; Sathekge et al., 2017), were found to be promising compounds for therapy of mCRPC. Nevertheless, PSMA-617 bearing the linker moieties 2-naphthyl-l-alanine and 4-(aminomethyl)cyclohexanecarboxylic acid still presents the standard reference regarding pharmacokinetics and internalization (Benesova et al., 2015; Benesova et al., 2016). Furthermore, DOTA tracers are also appropriate for labeling with some α-emitting isotopes, such as ^225^Ac and ^213^Bi. Until now, ^225^Ac-PSMA-617 is considered to be the compound to deliver the highest cumulative dose (McDevitt et al., 2001; Miederer et al., 2008; Wadas et al., 2014). As ^225^Ac decays though various pathways, themselves producing radioactive daughter nuclides, internalization is of special interest for ^225^Ac-therapy. Thus, novel ligands that offer increased internalization into tumor cells would present an advantage due to higher accumulation of radioactivity within the cell and prolonged retention of radioactive daughter nuclides. Therefore, the present study was focused on further optimization of PSMA-617. The preclinical characterization of four newly developed PSMA-617 analogues is described (Figure 1). For this purpose, derivatives of PSMA-617 with modified lipophilic properties were designed. Compounds containing aromatic building blocks were found to improve its properties; in particular, a benzyl residue placed within the lipophilic pocket of PSMA triggered cellular internalization and caused favorable pharmacokinetics by reducing the amount of tracer within the kidneys (Liu et al., 2008; Kularatne et al., 2009; Eder et al., 2012).

## Materials and Methods

The solvents and chemicals were obtained from Merck, Sigma-Aldrich, Iris Biotech, Macrocyclics, or CheMatech. The compounds were used without further purification.

### Synthesis of the Ligands

**FIGURE 2:**
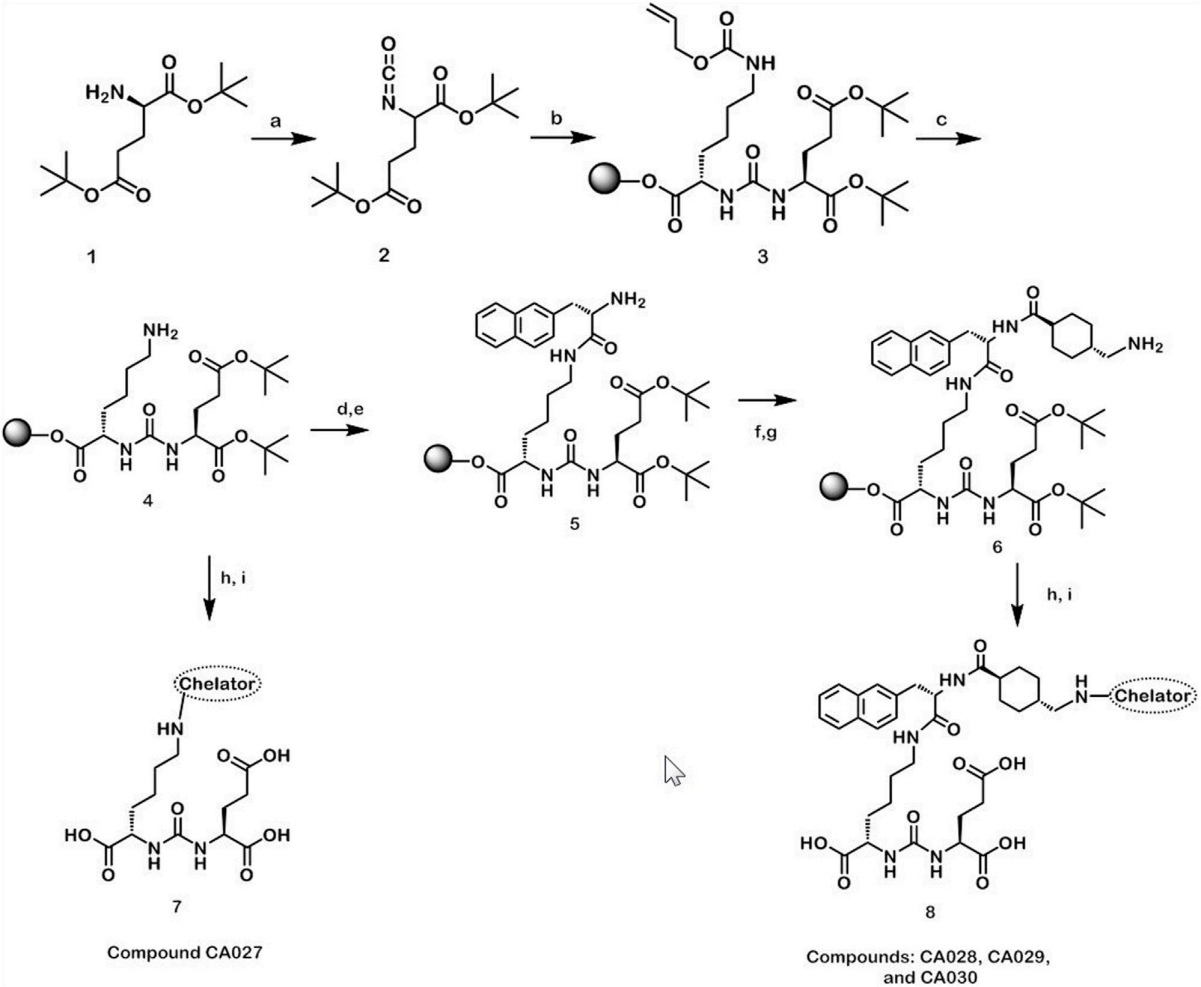
Reaction scheme for the synthesis of the novel PSMA ligands **(A)** triphosgene, diisopropylethylamine, dichloromethane, 0°C; **(B)** H-Lys (Alloc)-2CT-resin, dichloromethane; **(C)** Pd [P(C_6_H_5_)_3_]_4_, morpholine, dichloromethane; **(D)** Fmoc-2-Nal-OH, HBTU, diisopropylethylamine, DMF; **(E)** 50% piperidine, DMF; **(F)** trans-4-(Fmoc-aminomethyl)cyclohexanecarboxylic acid, ethyl-cyano (hydroxyimino)acetate, DIC, DMF; **(G)** 50% piperidine, DMF; **(H)** chelator, diisopropylethylamine, DMF; **(I)** 95% TFA, 2.5% triisopropylsilane and 2.5% H_2_O.

The synthesis of the four novel PSMA ligands is summarized in Figure 2. As previously described by Eder *et al.*, solid-phase peptide chemistry was applied to obtain the glutamate-urea-lysine binding motif (steps 1–4) and the linker (Eder et al., 2012) and Benešová et al. (Benesova et al., 2015). In brief, triphosgene was used in the first step of the synthesis for the formation of the isocyanate of the glutamyl moiety. A resin-immobilized (applying 2-chlorotrityl resin; Iris Biotech) ɛ-allyloxycarbonyl-protected lysine was reacted for 16 h. This resulted in the allyloxycarbony-protected urea binding motif. The allyloxycarbonyl-protecting group was cleaved after filtration of the resin. The respective chelator was attached to the resin previously linked to Fmoc-2-naphthylalanine and *trans*-4-(Fmoc-aminomethyl)-cyclohexanecarboxylic acid (see below). The final compound was cleaved from the solid support and analyzed by high-performance liquid chromatography (HPLC) and mass spectrometry. Subsequently, the compounds were purified by preparative HPLC. Using a linear gradient elution from 0.1% trifluoroacetic acid (TFA) in water to 0.1% TFA in acetonitrile, the compounds were separated on a Chromolith^®^ SemiPrep-column (Merck). The desired products were analyzed by HPLC with the eluent system described above within 5 min on a Monolith RP HPLC column (100 × 3 mm; Merck) and by liquid chromatography–mass spectrometry (LC-MS) (Thermo Fisher Scientific). Finally, the product fractions were pooled and lyophilized.

### Synthesis of the Chelator Moieties

The synthesis of the chelator 1-(4-methylbenzoic)-1,4,7,10-tetraazacyclododecane-4,7,10-triacetic acid for compound CA027 and CA028 was described by Kielar et al. (2018). The chelators *p*-NCS-benzyl-DOTA-GA (ligand CA029) and *p*-SCN-Bn-DOTA (ligand CA030) were purchased from CheMatech and Macrocyclics, respectively. Prior to coupling to the PSMA ligands, the chelators were characterized by LC-MS.

### Radiochemical labeling of ^68^Ga-CA028

^68^Ga was eluted from a^68^Ge/^68^Ga generator (iThemba LABS). To a mixture of 320 µL sodium acetate buffer (2.5 M in water, pH 4–5), 10 µL ascorbic acid (20% in water), and 400 MBq ^68^Ga in 0.6 M HCl, 20 nmol of the conjugate CA028 (1 mM in DMSO) was added to obtain a final pH between 3.6 and 4.2. After heating at 95°C for 5 min, the labeling was followed by radio-HPLC (0–100% ACN in 5 min, Monolith column, Merck) using a flow rate of 2 ml/min and a retention time of 2.4 min.

### Radiochemical Labeling of ^177^Lu-PSMA-CA028

Approximately 20 MBq [^177^Lu]LuCl_3_ (Isotope Technologies Garching) was initially mixed with 0.4 M sodium acetate buffer (200 µL of pH = 5). 40 µL of the solution was mixed with 2 µL of a 1 mM solution of the compound CA028 (in 10% DMSO in water) and 2 µL of a 20% (w/w) solution of ascorbic acid (in water) and heated to 95°C for 10 min. The labeling solution had a pH of approximately 5.0. The labeling was checked by radio-HPLC as described above.

### *In Vitro* Experiments

The *in vitro* experiments were conducted in triplicate. At least three independent sets of data were obtained for every experiment.

### Competitive Cell Binding Assay

The competition binding assay was performed using the cell line C4-2 (ATCC^®^ CRL-3314™; American Type Culture Collection). This is a subline of the PSMA-positive cell line LNCaP. The cells were cultivated in RPMI 1640 medium (PAN-Biotech), supplemented with 10% fetal bovine serum and 2 mM l-glutamine (PAN-Biotech) at 37°C and 5% CO_2_ in a humidified atmosphere.

A 96-well MultiScreen_HTS_-DV filter plate (Merck Millipore) was incubated at room temperature with 100 µL of a 1% bovine serum albumin (BSA) solution in PBS (phosphate-buffered saline) per well for 30 min. After removal of PBS/BSA solution, 1 × 10^5^ C4-2 cells in 50 µL of Opti-MEM medium (Gibco, Life Technologies) were seeded into each well. The inhibitory potency was determined using the ^68^Ga-labeled PSMA-HBED-CC dimer (^68^Ga-PSMA-10) (Schäfer et al., 2012) as the radioligand at a concentration of 0.75 nM. All non-labeled compounds were dissolved in Opti-MEM and added as competing ligands at several concentrations: 0, 0.5, 1, 2.5, 5, 10, 25, 50, 100, 500, 1,000, and 5,000 nM. After incubation at 37°C for 45 min, the cells were washed twice with PBS at 4°C. The radioactivity in the supernatant and that accumulated by the cells was determined with a γ-counter (Packard Cobra Auto-gamma). Using Graph Pad Prism 5.01 software, a nonlinear regression algorithm was calculated to obtain the 50% inhibitory concentration (IC_50_). These experiments were performed in quadruplicate.

### Determination of the Internalization Efficiency

The specific internalization ratio was determinated in 24-well plates. The plates were previously coated with 0.1% poly-l-lysine in PBS for 20 min and washed with PBS once. C4-2 cells (10^5^ cells per analysis) were added to each well and equilibrated overnight. The cells were incubated for 45 min with 250 µL of a 30 nM solution of the ^68^Ga-labeled compounds either in a water bath at 37°C or on ice. The PSMA-receptor-blocking agent 2-(phosphonomethyl)pentanedioic acid (2-PMPA; 500 μM; Axxora) was used to verify the specificity of the cellular uptake of the novel compounds. The cells were washed thrice (using 1 ml of ice-cold PBS), followed by an incubation with 0.5 ml of a glycine solution (50 mM in HCl at a pH of 2.8) for 5 min, washed once with 1 ml of ice-cold PBS, and finally lysed with 0.5 ml of 0.3 M NaOH. The lysate was collected, and the radioactivity was measured in a gamma-counter. Triplicate measurements were performed for all experiments.

### Serum Stability

After radiolabeling of the compounds, serum stability was examined by HPLC and ITLC (instant thin-layer chromatography). 50 µL (20 MBq) of the ^177^Lu-labeled ligands was added to 200 µL human serum (Sigma-Aldrich) and incubated at 37°C. At different time points (0, 2, 24, 48, and 72 h) 0.5 µL was applied onto iTLC-SG-glass microfiber chromatography paper strips (5 × 0.5 cm; Agilent Technologies) at a distance of 1 cm from the bottom. The strip was placed upright into a vial containing 120 µL of sodium citrate buffer (0.5 M, pH = 5.0). The buffer front was allowed to rise to the top. Subsequently, each strip was cut in eight equally sized pieces that were subsequently measured in a gamma-counter. For HPLC analysis, an equal volume of ACN was added to the samples; under these conditions, the serum proteins precipitated. The samples were centrifuged for 10 min at 12,000 × g. Following the separation of the pellet and the supernatant, their relative activity (%) was determined. An aliquot of the supernatant was analyzed by radio-HPLC (0–100% ACN in 5 min, Monolith column, Merck), with a flow rate of 2 ml/min.

### Organ Distribution Animal Experiments

Athymic male nude mice (BALB/c nu/nu) were purchased from Charles River at 4‒5 weeks of age (19–23 g). The mice were kept under pathogen-free conditions for 1 week prior to the study. The mice had free access to water and food and were housed with a 12-h/12-h light/dark cycle. The xenografts required for positron emission tomography (PET) imaging and biodistribution studies were obtained by inoculation of 5 × 10^7^ C4-2 cells in 50% Matrigel in Opti-MEM I (1 ×) medium. For this purpose, the mice were anesthetized with 2% sevoflurane, and inoculation was performed subcutaneously on the right trunk. When the size of the tumor was approximately 1 cm^3^, the studies were performed.

### Determination of Plasma Half-Life

The radioligands ^68^Ga-CA027 and ^68^Ga-CA028 (4–6 MBq) were injected into athymic nude mice (n = 3) via the lateral tail vein. Blood (25–50 μL) was collected 2, 5, 15, 30, 45, 60, 90, and 120 min post injection via either the tail or saphenous vein, and the activity of the blood samples was determined in a γ-counter. Both procedures furnished consistent and reproducible results. The percentage of the injected dose per gram of tissue (%ID/g) was calculated, and the resulting values were plotted as a function of time. A biexponential decay curve was used as a fit function, accounting for a short blood half-life (a-phase) during the initial phase directly after injection and a longer blood half-life during the terminal phase (b-phase). The data analysis program Prism 7™ was used to determine both half-lives, t_1/2-a_ and t_1/2-b_, as well as their relative contribution (in %) to the overall half-life (t_1/2_). Subsequently, both half-lives were weighed according to their relative contribution to the overall blood half-life t_1/2_. At the time of tracer injection (t = 0), it was assumed that %ID/g = 50. The injected dose at t = 0 equals 100%, divided by the total mass of blood (approximately 2 g in the case of 6‒8 weeks old mice) gives %ID/g _t=0_ = 50.

### *In Vivo* Fate of 177Lu-CA028 and 177Lu-PSMA-617

The metabolization of the radiolabeled tracers *in vivo* was studied by radio-HPLC analysis. Male nude C4-2 tumor-bearing mice (n = 3) were injected with ^177^Lu-CA028 or ^177^Lu-PSMA-617 (10 MBq, 0.2 nmol in approximately 100 µL of 0.9% saline) via the tail vein. Ten min post injection, blood, the liver, and the kidneys were dissected. The tissues were rinsed with ice-cold saline, blotted dry, treated with 2 ml of 0.1 M NH_4_OAc/EtOH (35:65), and homogenized with an Ultra-Turrax T8 (IKA Labortechnik). The samples were then centrifuged for 10 min at 12,000 × g and 4°C. For HPLC measurements, an aliquot of the supernatants was prepared by precipitation of the proteins with ACN as described above for serum stability testing. The samples were analyzed by radio-HPLC (0–100% ACN in 5 min, 2 ml/min, Monolith column, Merck). The fractions were collected every 10 s over the whole course of the chromatographic run. Subsequently, the activity of the samples was measured in a gamma-counter to reconstruct a chromatogram.

### Biodistribution Studies

Based on the results of the PET imaging, ^68^Ga-CA028 and ^68^Ga-CA030, the most promising compounds, were chosen for biodistribution studies in C4-2 tumor-bearing mice. The labeled compound (0.05 nmol, 20 MBq) was administered to the mice via tail vein injection. Animals were sacrificed and organs were harvested at the following time points: 20 min, 1, 2, and 4 h *p.i*. The organs were dissected and weighed, the activity was measured with a γ-counter, and the % ID/g was calculated.

### Dynamic and Static PET Imaging

For small-animal PET imaging, the different ^68^Ga-labeled PSMA ligands (0.5 nmol, 20 MBq in approximately 100 µL 0.9% saline) were injected into C4-2 tumor bearing mice. The dynamic PET was recorded in a small animal PET scanner (Siemens Inveon D-PET). The standardized uptake values (SUVs) were obtained from conventional (non-dynamic) PET images. The formula for the SUV was $\text{SUV}=\frac{\mathrm{activity}\;\mathrm{in}\;\mathrm{ROI}\;\left(\frac{\mathrm{Bq}}{\mathrm{ml}}\right)\;\times \;\mathrm{animal}\;\mathrm{weight}\;\left(g\right)}{\mathrm{injected}\;\mathrm{dose}\left(\mathrm{Bq}\right)}$

Manual delineation of the respective appropriate whole organ (heart, kidneys, bladder, tumor, with an approximate volume of 100–500 µL) yielded the volumes of interest (VOIs). For this purpose, whole organs or parts of the organ/tissue (liver and muscle) were used. The images were reconstructed based on the procedure: OSEM 3D/SP MAP with 16 subsets, two iterations, and an image x-y size: 256, image z size: 161. The data were not modified with a post-processing filter. Analysis of the images and time-activity curves (TACs) was conducted on the Inveon™ Acquisition Workplace (IAW) from Siemens IRW 4.1. Dynamic PET scans were performed 0–60 min *p.i.*, and images were reconstructed in three time frames of 20 min (0–20 min, 20–40 min, and 40–60 min) for visual display. A static PET scan was generated at 1 h post injection. The mean SUVs were plotted over time in order to compare the different radiotracers.

### PSMA-PET/CT of Prostate Cancer Patients

Diagnostic PSMA-PET/computed tomography (CT) examinations were performed 1 and 3 h after antecubital injection of 339 MBq/20 nmol ^68^Ga-CA028 or 295 MBq/20 nmol ^68^Ga-CA030 per patient, respectively. The method for assessing the biodistribution was performed as previously described by Afshar-Oromieh *et al.* (Afshar-Oromieh et al., 2015b). Applying the clinical standard software Syngo (Siemens), which was used to define the VOIs in the PET images, the activity distributions of the source organs was determined. This reference (Afshar-Oromieh et al., 2015b) was also used as a standard of reference for ^68^Ga-PSMA-617 (Table 3).

## Results and Discussion

### Synthesis of the Ligands

The solid-phase synthesis of the binding motif containing the linker moieties 2-naphthyl-l-alanine and cyclohexanecarboxylic acid was conducted on a 2-chlorotrityl-resin, followed by the coupling of the chelator. The conjugates could be obtained in yields of approximately 40% after cleavage from the resin and purification by HPLC. The purity was equal or above 95%. The characteristics of the synthesized compounds are summarized in Table 1.

**TABLE 1:** Analytic data of the novel ligands.

Compound	Molecular weight (g/mol)	[^68^Ga-ligand]-HPLC retention time (min)	m/z^a^ experimental
CA027	781.35	1.55	782.33
CA028	1117.53	2.37	1118.53
CA029	1206.52	2.60	1207.52
CA030	1277.56	2.61	1278.56

a Mass spectrometry of non-labeled ligands detected as [M + H]^+^.

### Competitive Cell Binding Internalization Ratios and Serum Stability

The results of the *K*_i_ determination revealed nanomolar binding affinities of the synthesized ligands to PSMA. As shown in Table 2, among all new compounds, CA030 revealed the highest affinity to PSMA, followed by CA029 and CA028. CA027, which contains none of the two linkers, had the lowest affinity to PSMA.

**TABLE 2:** PSMA Inhibition Potencies (Expressed in *K*_i_ values) and Specific Internalization Values.

Compound	*K*_i_ (nM)	Specific cell surface (%IA/10^6^cells)	Specific lysate (%IA/10^6^ cells)
CA027	33.82 ± 2.13	2.18 ± 0.50	1.70 ± 0.24
CA028	15.17 ± 6.11	94.12 ± 2.70	41.22 ± 2.72
CA029	11.04 ± 4.87	46.83 ± 3.07	44.26 ± 3.96
CA030	4.79 ± 1.31	43.46 ± 1.50	53.78 ± 5.45
PSMA-617	2.34 ± 2.94	45.18 ± 3.61	15.55 ± 3.07

Data are mean ± SD (*n* = 3).

The lipophilicity of the spacer is probably the key factor of highly efficient PSMA binders that follow the basic concept [urea motif]—[spacer]—[chelating moiety]. The lipophilicity accounts for the efficiency of PSMA-617 as compared to alternative tracers. On the other hand, the PSMA-tracer interaction is a multifactorial interaction, which has to take further interactions *in vivo* into account.

The straightforward further increase of the lipophilicity, as performed in albumin binding PSMA tracers, did not lead to compounds with an overall improvement.

As the compounds were analyzed by reversed phase HPLC, an alternative measure for lipophilicity as compared to octanol water partition coefficients, this hypothesis could be checked. In general, the affinity goes along with the lipophilicity. However, the fact that CA029 to CA030 show similar retention times, while CA030 has a significantly improved affinity, shows the limit of this factor.

Moreover, the data were analyzed with respect to a possible correlation between the distances between the urea motif and the chelating moiety. This may be part of the multifactorial interaction of the tracers as the spacer length accounts for the positioning of the lipophilic moiety of the spacer with respect to the anticipated lipophilic binding site in the binding pocket. As a precise prediction of the intramolecular distance would require complex NMR analyses or molecular modeling studies, the number of atoms does provide a reasonable estimation of the spacer length. In contrast to the dependency observed for the lipophilicity, the spacer length did not turn out to provide a clear correlation to the affinity.

With the exception of ^68^Ga-CA027, the novel compounds showed higher specific internalization in C4-2 cells than the reference, for example, 53.78% ID/10^6^ cells for CA030 vs. 15.6% ID/10^6^ cells for PSMA-617 (Table 2).

The stability of the compounds radiolabeled with ^68^Ga and ^177^Lu was studied in human serum. As indicated by HPLC and radio-ITLC, the ^68^Ga-labeled compounds did not show degradation after incubation for 2 h in human serum. In contrast to ^177^Lu-CA029, ^177^Lu-CA030, and the reference compound ^177^Lu-PSMA-617, which showed no release of ^177^ Lu, analysis of ^177^Lu-CA028 revealed 40% of free activity at this time point (Supplementary Figure S1).

### *In Vivo* and *Ex Vivo* Experiments

**FIGURE 3:**
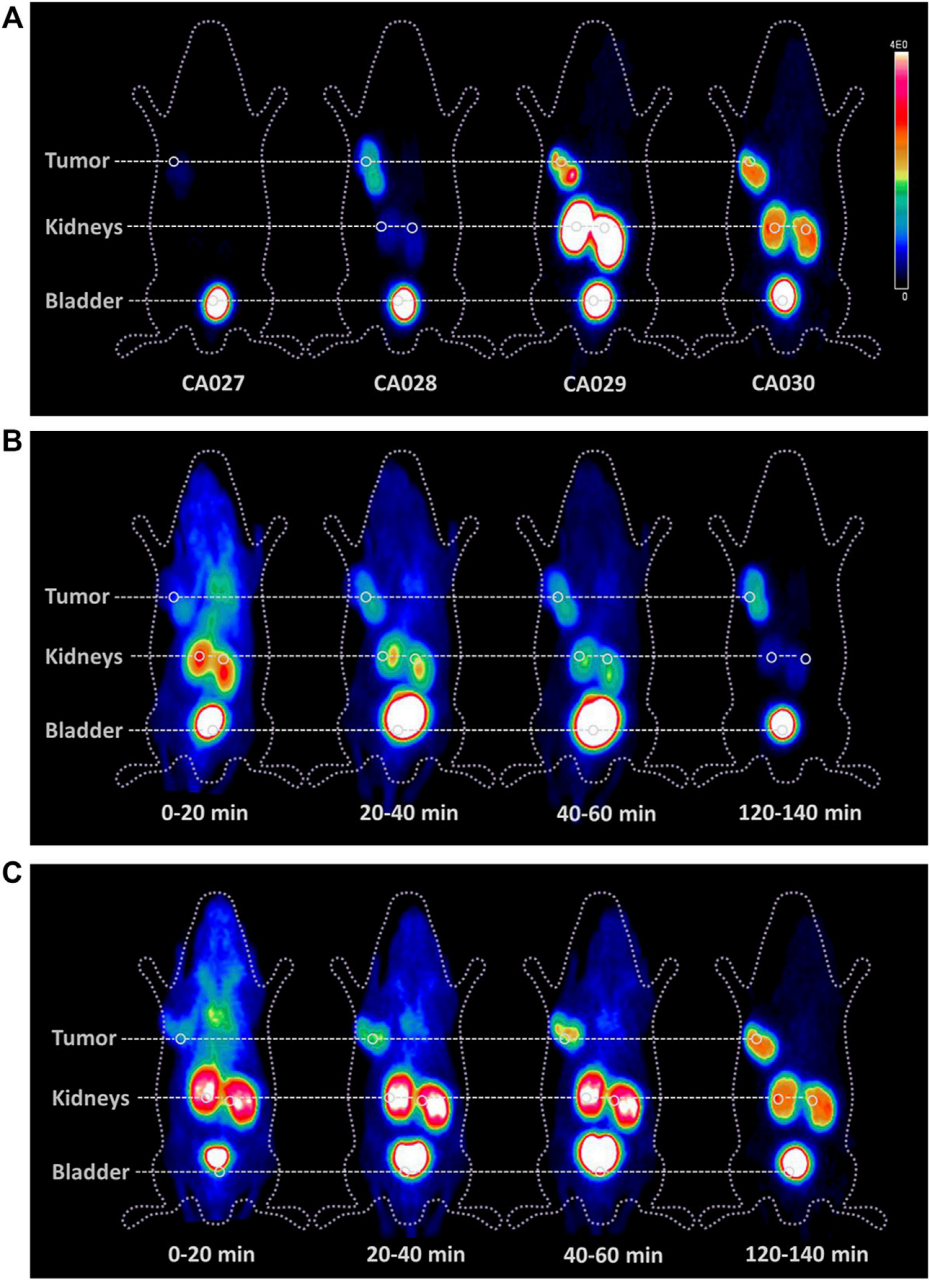
Comparison of whole body small animal PET scans as maximum-intensity projections of BALB/c nude mice bearing C4-2 tumor xenografts. PET imaging of the four new PSMA ligands radiolabeled with ^68^Ga (20 MBq; 0.2 nmol) 2 h post injection **(A)** and the time courses of ^68^Ga-CA028 **(B)** and ^68^Ga-CA030 **(C)**. The color bar gives a link between the SUV and the color scale of the PET image with 0 = minimum and 4E0 = maximum.

The metabolization of ^177^Lu-CA028 *in vivo* was studied in comparison with ^177^Lu-PSMA-617 by radio-HPLC analysis. Radio-HPLC chromatograms of extracts from the kidney, blood, liver, and tumor confirmed that the activity elutes at the retention time of the intact tracer (Supplementary Figure S2). Therefore, the integrity of the complex of both ligands is proven within the main *in vivo* distribution period. The time–activity curves and the mean SUV body weight values generated from the dynamic PET imaging of ^68^Ga-CA028 1 h *p.i.* demonstrated a tumor-to-kidney ratio of 2.08 (Supplementary Figure S3). At 2 h *p.i.*, this ratio was increased to 2.69 (Figure 3; Supplementary Table S1), while the ligands ^68^Ga-CA029 and ^68^Ga-CA030 showed lower tumor-to-kidneys ratios of 0.52 and 0.33, respectively (Figure 3, Supplementary Figures S4, S5).

**FIGURE 4:**
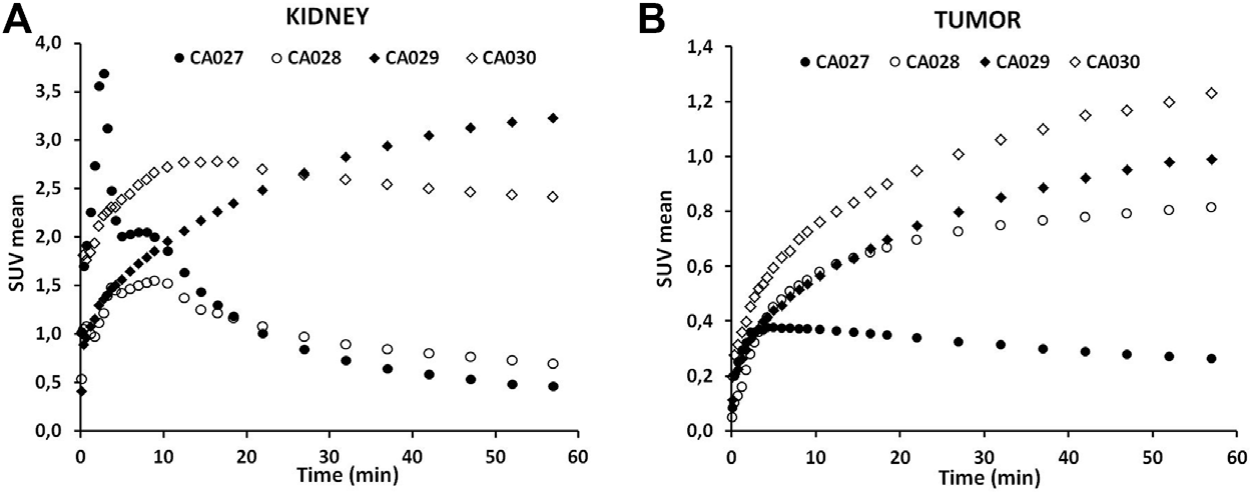
Time–activity curves of the novel PSMA ligands labeled with the ^68^Gafor kidney **(A)** and for the tumor **(B)** up to 1 h post injection. Data are mean SUVs.

The time–activity curves revealed a fast clearance of the tracer—^68^Ga-CA028 showed a high tumor accumulation and high kidney values. In contrast, for ^68^Ga-CA027, a faster clearance by the kidney at a tumor accumulation was found (Figure 3). Even though ^68^Ga-CA030 demonstrated higher kidney uptake values than ^68^Ga-CA028, it showed the highest tumor uptake among all compounds (Figure 4B). Therefore, ^68^Ga-CA028 and ^68^Ga-CA030 were chosen for detailed investigation. The small animal PET images in Figure 3A demonstrated a very fast kidney clearance and low tumor accumulation of ^68^Ga-CA027. Distinctly, the radiotracers ^68^Ga-CA028, ^68^Ga-CA029, and ^68^Ga-CA030 showed high tumor accumulation (Figure 4B). ^68^Ga-CA029 showed the highest kidney uptake, followed by ^68^Ga-CA030 (Figure 4A). In order to analyze the pharmacokinetic parameters such as circulation time of the tracer in the blood, the plasma half-life determination of ^68^Ga-CA027 and ^68^Ga-CA028, the tracers with the lowest kidney uptake, was performed. ^68^Ga-CA027 showed a comparably short plasma half-life of t_1/2_ = 2.7 min, and ^68^Ga-CA028 showed a longer circulation with t_1/2_ = 5.2 min (Supplementary Figure S4).

**FIGURE 5:**
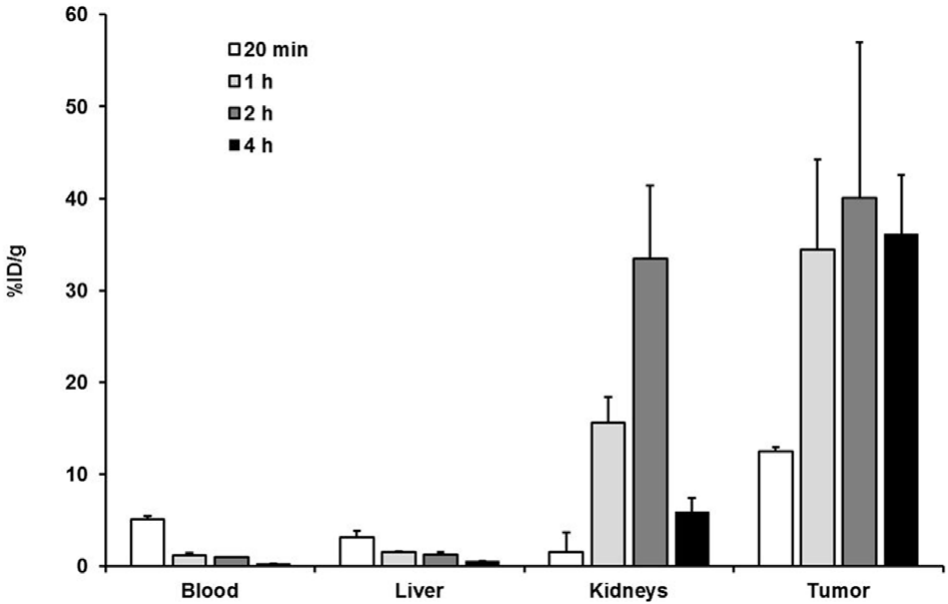
Organ distribution of ^68^Ga-CA028 (0.05 nmol, 20 MBq) expressed as % ID/g of tissue ±SD (*n* = 3) at 20 min and 1, 2, and 4 h post injection.

**FIGURE 6:**
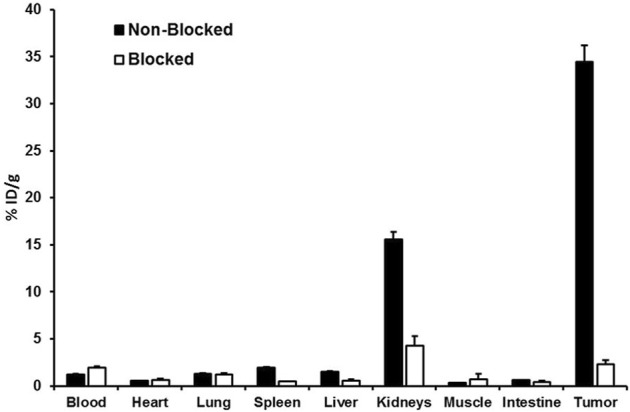
Blockade experiment with ^68^Ga-CA028 (0.05 nmol, 20 MBq) 1 h post injection. 2 mg of PSMA-617 per kilogram of body weight was injected at the same time as ^68^Ga-CA028. Data are mean expressed (*n* = 4).

The results obtained for the biodistribution of the promising PSMA ligands ^68^Ga-CA028 (Figure 5; Supplementary Table S2) and ^68^Ga-CA030 (Supplementary Figure S4) are in line with the PET imaging. The graphics (Figure 5, Supplementary Figure S5) show the organ distribution at the time points of 20 min and 1, 2, and 4 h *p.i*. For ^68^Ga-CA028, a tumor uptake of 12.53 ± 0.45 %ID/g 20 min after injection was observed. 1 h *p.i.*, the amount of the tracer accumulation in the tumor was higher than that in the kidneys. The specificity of the binding to PSMA was proven with a blockade experiment: co-injection of non-labeled PSMA-617 [2 mg/kg] led to a strong decrease of accumulated ^68^Ga-CA028 in C4-2 tumors (34.46 ± 9.76 %ID/g to 1.31 ± 0.34 %ID/g) and in the kidneys (15.58 ± 2.79 %ID/g to 2.37 ± 0.52 %ID/g) 1 h *p.i.* (Figure 6).

### PSMA-PET/CT of Prostate Cancer Patients and Imaging of ^68^Ga-CA028 and ^68^Ga-CA030

**FIGURE 7:**
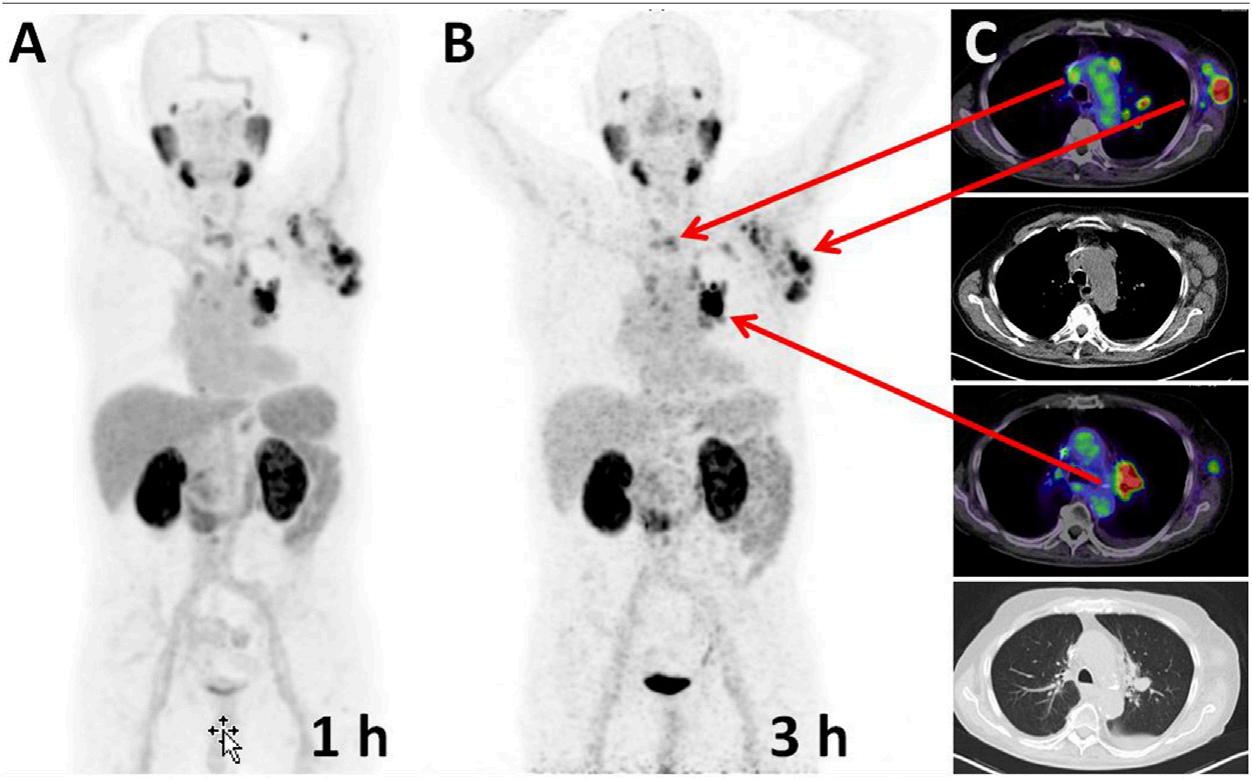
Maximum intensity projections of a PSMA-PET of a prostate cancer patient performed 1 h **(A)** and 3 h **(B)** post injection of 339 MBq/20 nmol ^68^Ga-CA028. **(C)** Cross-sectional slices demonstrate axillary and hilar lymph node metastases (red arrows), also delineable on the correlated CT which serves as a standard of reference.

**FIGURE 8:**
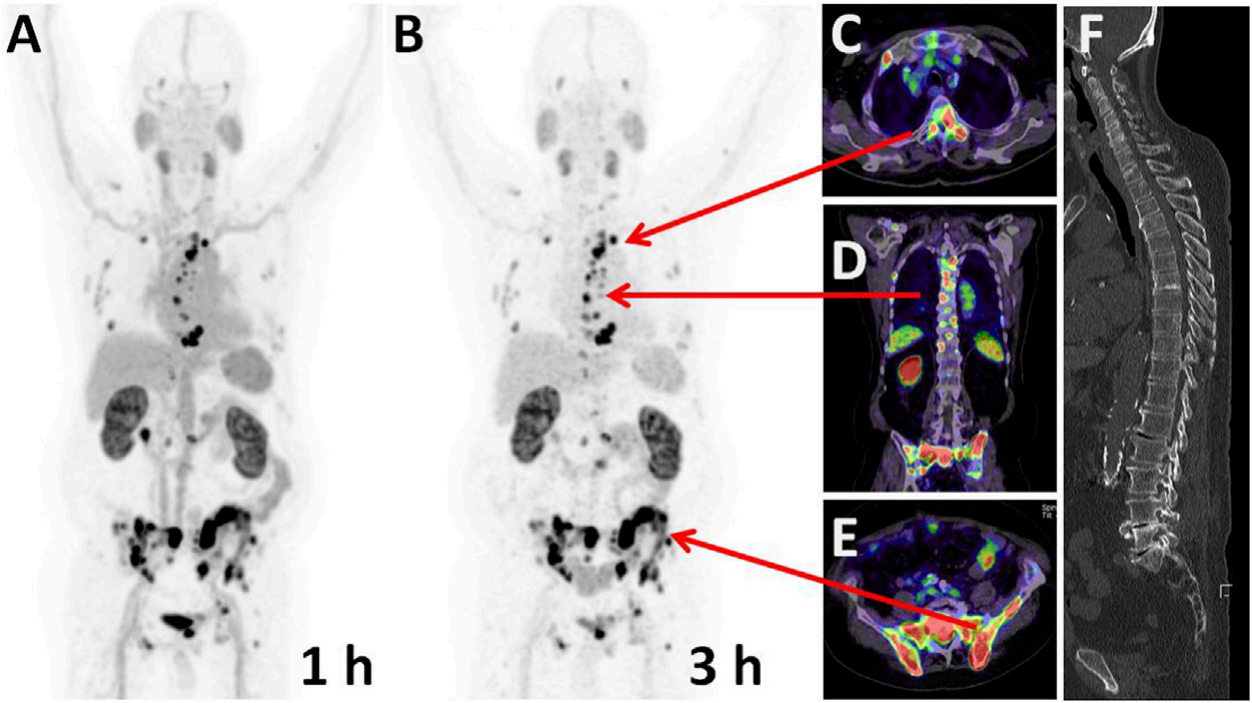
Maximum intensity projections of a PSMA-PET of a prostate cancer patient performed 1 h **(A)** and 3 h **(B)** post injection of 295 MBq/20 nmol ^68^Ga-CA030. Arrows point to the position of the cross-sectional slices demonstrating bone metastases in multiple regions of the axial skeleton **(C–E) (F)** In the CT, no typical osteoblastic reactions allowed tumor delineation by morphological information alone.

In order to prove the clinical applicability of ^68^Ga-CA028 and ^68^Ga-CA030, PET/CT imaging in first patients was performed. The resulting images are illustrated in Figures 7, 8. The imaging with ^68^Ga-CA028 and ^68^Ga-CA030 confirmed the results obtained *in vitro* and in the animal models. The PET scans and the SUVs were acquired with standard scanner settings, calibrated for pure positron emitters (23). The SUV values obtained at 1 versus 3 h *p.i.* are presented in Table 3. As reflected by these values, a high and stabile accumulation in the tumor is achieved.

**TABLE 3:** Safety dosimetry of diagnostic SUV mean values of ^68^Ga-CA028, ^68^Ga-PSMA-617 [values from Afshar-Oromieh et al. (2015a) and Afshar-Oromieh et al. (2015b)], and ^68^Ga-CA030 and based on an adult male phantom in OLINDA.

Tissue	^68^Ga-CA028		^68^Ga-PSMA-617		^68^Ga-CA030	
	1 h	3 h	1 h	3 h	1 h	3 h
Lacrimal gland	7.4	7.6	4.9	5.9	7.1	9.0
Nasal mucosa	2.9	3.5	2.9	3.4	3.3	3.8
Parotid gland	11.0	8.0	10.4	13.1	6.1	6.7
Submandibular gland	14.4	8.9	10	12.4	8.7	11.3
Sublingual gland	3.8	2.8	4.6	4.0	4.1	4.9
Blood pool, mediastinal	3.2	2.9	2.5	2.4	5.6	4.5
Liver	4.3	1.8	3.3	2.7	5.8	5.9
Spleen	5.1	2.5	4.3	3.5	7.2	6.1
Proximal small intestine	8.4	10.4	4.7	5.5	4.1	5.0
Colon	4.9	4.2	3.5	4.0	4.9	4.7
Kidneys	13.8	10.9	15.6	17.0	13.4	16.5
Gluteal muscle	0.7	0.4	0.7	0.7	0.8	0.7
Bone metastasis	4.5	4.1	9.4	6.3	27.2	37.7
Lymph node	13.5	10.7	7.1	13.5	—	—
Bone metastasis	6.9	5.7			30.9	32.6
Bone metastasis	4.6	4.4			31.0	33.1
Lymph node	17.0	25.4			—	—
Lymph node	5.0	4.1			—	—
Lung metastasis	5.7	4.7			—	—

The main purpose of this study was the synthesis, the preclinical development, and a first clinical proof of novel ^68^Ga/^177^Lu-labeled PSMA ligands. The rationale for the design was the improvement of the linker of PSMA-617. The novel compounds comprise the Lys-NH-CO-NH-Glu binding motif (Chen et al., 2008; Hillier et al., 2009) as their basic structure. The linker moiety was based on the linker used in PSMA-617 (Zhang et al., 2010; Benesova et al., 2016). This linker and the DOTA moiety were fine-tuned to improve the internalization efficiency and the tumor to background ratio. Four representative PSMA binding molecules, CA027, CA028, CA029, and CA030, are described here. In CA027, omission of the linker consisting of 2-naphthylalanine and cyclohexanecarboxylic acid substantially decreased the affinity, internalization, and consecutive tumor uptake. CA029 was found to strongly accumulate in the tumor; however, due to a high kidney accumulation, it was considered inferior to PSMA-617. CA028 and CA030 showed high tumor uptake and lower kidney retention and were consequently studied in greater detail.

As ^68^Ga-CA028 showed a rapid clearance from the kidneys, this compound reached a high tumor to kidney ratio and its characteristics might be ideally for diagnostic application (Figure 7). However, after recent introduction of ^18^F-labeled diagnostic compounds such as ^18^F-PSMA-1007, the clinical demand for improved ^68^Ga-labeled diagnostics is shrinking (Giesel et al., 2017; Kesch et al., 2017). Since ^177^Lu-CA028 showed signs of degradation in human serum, its stability *in vivo* was analyzed by radio-HPLC of tissue extracts, which showed exclusively the intact tracer. However, in contrast to DOTA conjugates, the chelator moiety of CA028 lacks one amide bridge and thus slightly decreased complex stability with larger metals such as ^177^Lu and also ^225^Ac (not presented here) in comparison to ^68^Ga. Nevertheless, considering the rapid tumor targeting and internalization of these small molecules, the ligand might still have potential because some intra-cellular nuclide loss might be tolerated and the mean standard uptake values (mSUVs) derived from PET demonstrated a higher tumor-to-kidney ratio for CA028 in comparison with the other compounds tested. However, as shown in Table 2 and Figure 4, due to its higher internalization, ^68^Ga-CA030 reached a more persistent tumor uptake, which might be preferable for therapeutic applications, especially when used in combination with radionuclides that decay through unstable daughter nuclides (e.g., ^225^Ac).

The linker moieties 2-naphthyl-l-alanine and 4-(aminomethyl) cyclohexanecarboxylic acid used in PSMA-617 had already been proven to provide beneficial pharmacokinetics (Benesova et al., 2015; Benesova et al., 2016). The linker modification with the respective benzyl moiety connected to the DOTA chelator was found to further improve the tumor targeting characteristics, and in CA027, CA029, and CA030, further fine tuning was accomplished by using DOTA variants which contained reduced numbers of polar side chains. More lipophilic chelators were found promising to improve pharmacokinetics when they were introduced to realize copper (34) or lead (35) complexed PSMA ligands (Persson et al., 2013; Hao et al., 2017; Dos Santos et al., 2019; Dos Santos et al., 2020). However, it is relevant to emphasize that the presence of the benzyl group connected directly or via a linker to the chelator can be assumed to interact with the rigid part of the PSMA binding pocket. It has been demonstrated that the active binding site of PSMA is composed of two structural motifs, one representing a lipophilic pocket and the other interacting with urea-based inhibitors (Kularatne et al., 2009; Banerjee et al., 2019). Choosing optimal chelators enables an optimal size, flexibility, and polarity of the compounds (Antunes et al., 2007; Kane, 2010; Zhang et al., 2010). In total, the affinity of ^68^Ga-CA028 and ^68^Ga-CA030 to PSMA are comparable to that of ^68^Ga-PSMA-617 and ^68^Ga-PSMA-11 but presents improved internalization as demonstrated in our head-to-head-comparison (Table 2) or compared to the literature (Eder et al., 2014; Benesova et al., 2015). In the first clinical application (Figures 7, 8), the quantitative evaluation of ^68^Ga-CA028 and ^68^Ga-CA030 demonstrated a slightly prolonged circulation in the blood pool when compared to ^68^Ga-PSMA-617 (Table 3), similar but less pronounced to that of dedicated albumin binding PSMA tracers recently described (Benesova et al., 2018; Kuo et al., 2021). The available human data for ^68^Ga-labeled CA028/CA030 are limited to the initial distribution phase up to 3 h *p.i*. During this phase, the kidney uptake is dominated by an unspecific clearance. However, PSMA is also specifically expressed in kidneys and salivary glands (*3*), and it is possible that due to increased PSMA-specific internalization, the novel compounds could also be retained in the kidneys and the salivary glands at later time points (Baranski et al., 2019; Felber et al., 2021). Thus, dosimetry of ^177^Lu-CA028/30 up to several days *p.i.* would be required to draw a final decision whether the therapeutic range (ratio of absorbed dose to tumor vs. dose limiting normal organs) is really improved with the new compounds. As CA028 is hampered by its lower complex stability with larger radiometals and CA030 presents even more rapid internalization, which is considered favorable for labeling with ^225^Ac (currently considered one of the academically most interesting therapeutic radiometals), we propose CA030 as the most promising ligand for further clinical evaluation.

## Conclusion

The prostate-specific radiotracers ^68^Ga-PSMA-11, ^18^F-PSMA-1007, and ^177^Lu-PSMA-617 have recently improved the diagnostics and treatment of prostate cancer. ^68^Ga-CA028 revealed promising targeting characteristics for diagnostic applications.

However, to prove whether a real diagnostic advantage on the improved targeting clinical application is evident, further detailed evaluation is warrant. Due to more rapid tumor-cell internalization and faster non-PSMA-related kidney clearance in comparison to PSMA-617, CA030 presents a valuable novel ligand for further evaluation, for example, in combination with ^225^Ac. However, further clinical studies with long half-life nuclides are mandatory to investigate its pharmacokinetics beyond the 3 h interval evaluable with ^68^Ga-PET.

## Disclosure

Uwe Haberkorn, José Carlos dos Santos, Walter Mier, Clemens Kratochwil, Ulrike Bauder-Wüst, Martin Schäfer, and Klaus Kopka are inventors of the novel compounds described herein and have filed a patent regarding their synthesis and application (European patent application number/Patent number: 18197704.2/1109, date: 21. November 2018. Applicant/Proprietor: Universitätsklinikum Heidelberg). No other potential conflicts of interest relevant to this article were reported.

## Data Availability

The original contributions presented in the study are included in the article/Supplementary Material, and further inquiries can be directed to the corresponding author.
