# Supplementary material for: Refined Chelator Spacer Moieties Ameliorate the Pharmacokinetics of PSMA-617
Source: Front Chem. 2022 Aug 9;10:898692. doi: 10.3389/fchem.2022.898692 (PMC9396645; doi:10.3389/fchem.2022.898692)
Supplement: Supplementary file 1 [file DataSheet1.docx]

**SUPPLEMENT**

*In vivo* Metabolite Analysis


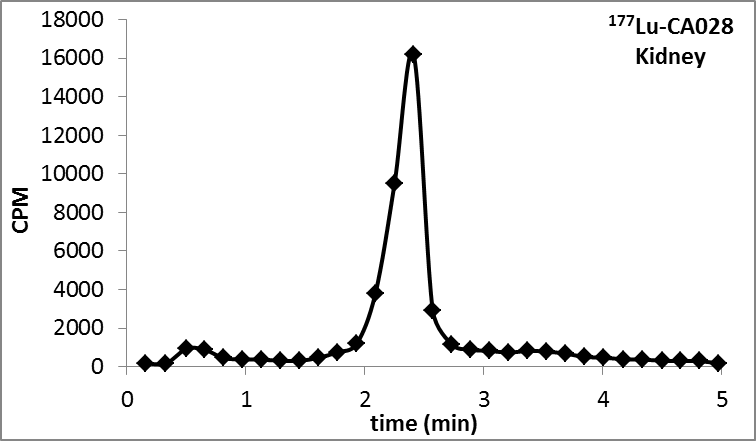

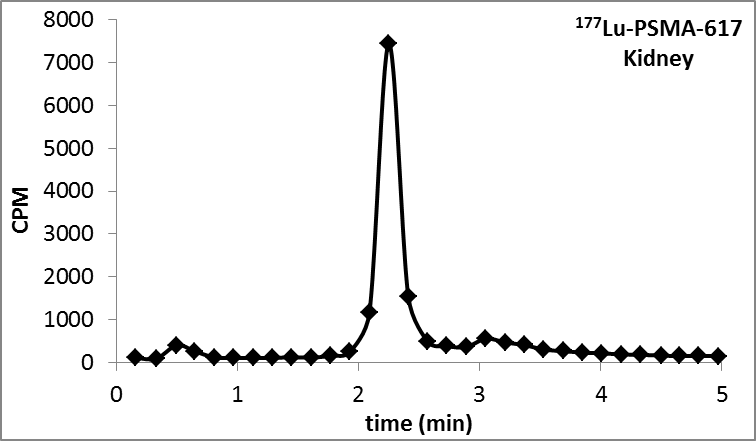


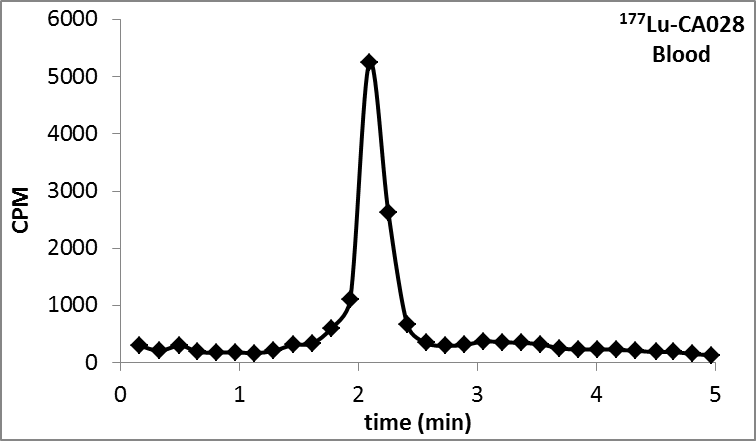

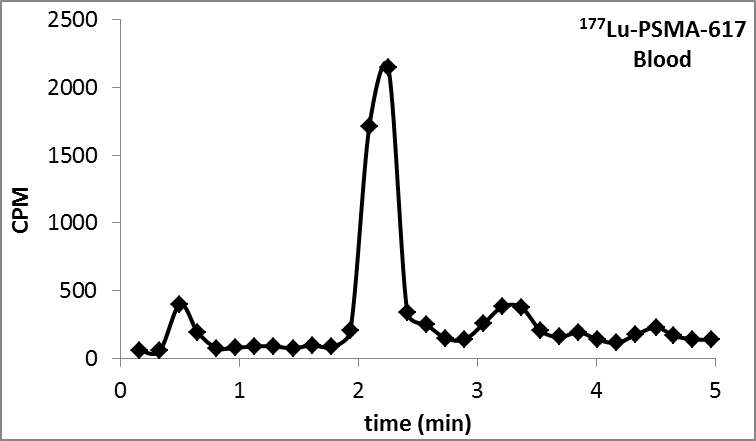


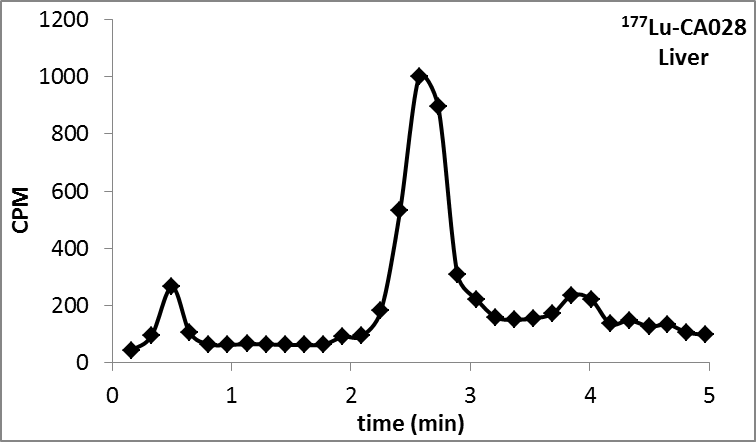

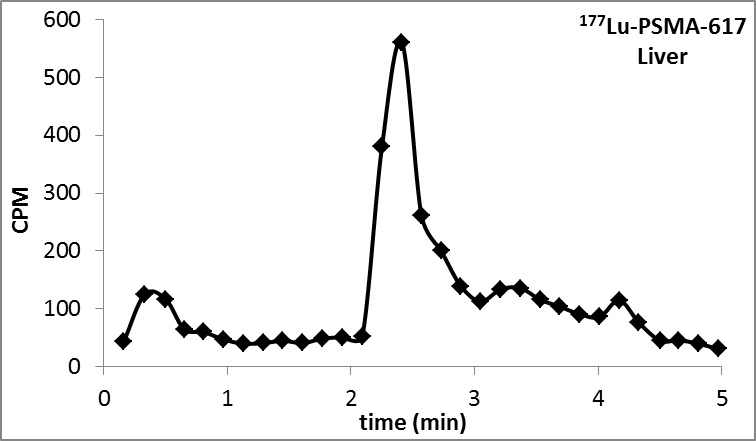


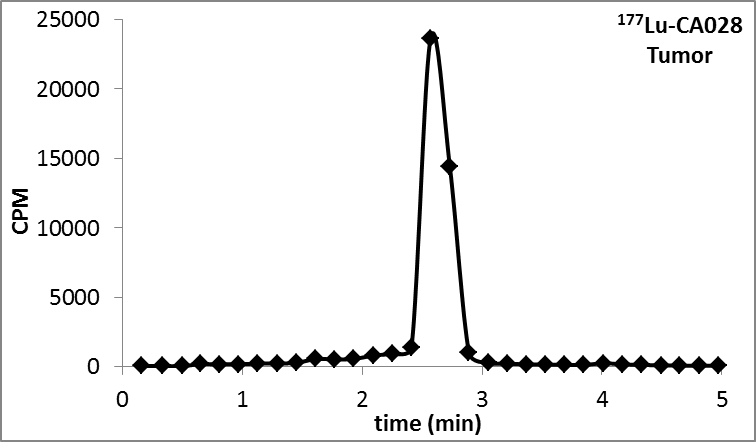

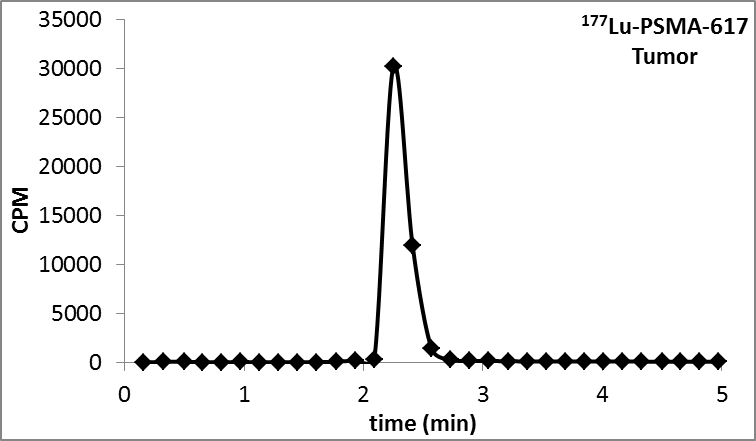


**Figure S1**. *In vivo* metabolite analysis of ^177^Lu-CA028 in comparison with ^177^Lu-PSMA-617 (10 MBq, 0.2 nmol in approximately 100 µl of 0.9% saline) in a BALB/c nude mouse (tumor) at 1 h *p.i*.. Radio-HPLC chromatograms of extracts from the kidney, the blood, the liver and tumor show that the activity elutes at the retention time of the intact tracer. This proves the integrity of the complex within the main distribution period.

**
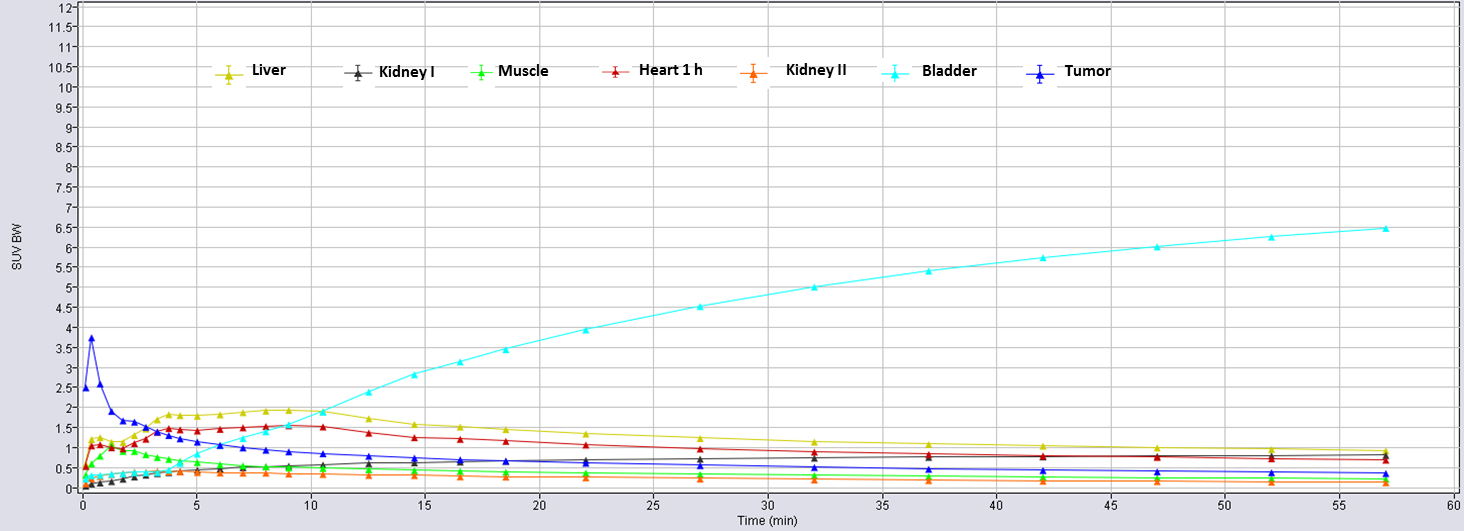
**

**Figure S2.** Time-activity curves for tumor and relevant organs up to 1 h post injection of 0.2 nmol (100 µL -as injected volume of 0.9% saline) of ^68^Ga-CA028 in a BALB/c *nu/nu* mouse bearing a C4-2 tumor xenograft. Data are mean standardized uptake value based on body weight-values (SUV_BW_).

**TABLE S1**

Mean standardized uptake values (mSUV) derived from the time-activity curves from small-animal PET of ^68^Ga-PSMA-CA028 in a BALB/c nu/nu mouse bearing a C4-2 tumor xenograft.

| mSUV | Heart | Liver | Kidneys | Bladder | Tumor | Muscle |
| --- | --- | --- | --- | --- | --- | --- |
| T_1_ = 1 h | 0.36 | 0.22 | 0.39 | 6.50 | 0.81 | 0.13 |
| T_2_ = 2 h | 0.13 | 0.09 | 0.29 | 2.5 | 0.78 | 0.05 |

**TABLE S2**

Organ Distribution for ^68^Ga-PSMA-CA030 in Tumor Bearing Mice

| Tissue | 20 min | | 1 h | 2 h | 4 h |
| --- | --- | --- | --- | --- | --- |
| Blood | | 4.33 ± 0.51 | 3.59 ± 1.52 | 1.42 ± 0.61 | 0.39 ± 0.14 |
| Heart | | 1.29 ± 0.30 | 1.03 ± 0.36 | 0.40 ± 0.09 | 0.16 ± 0.05 |
| Lung | | 2.63 ± 0.89 | 2.74 ± 0.72 | 1.08 ± 0.42 | 0.34 ± 0.10 |
| Spleen | | 8.37 ± 0.47 | 2.86 ± 1.86 | 1.39 ± 0.47 | 0.48 ± 0.09 |
| Liver | | 1.04 ± 0.18 | 1.52 ± 0.61 | 0.81 ± 0.15 | 0.50 ± 0.14 |
| Kidneys | | 63.89 ± 5.72 | 46.39 ± 23.44 | 20.19 ± 7.92 | 6.17 ± 0.82 |
| Muscle | | 1.39 ± 1.30 | 0.63 ± 0.21 | 0.28 ± 0.03 | 0.17 ± 0.02 |
| Small intestine | | 1.00 ± 0.38 | 0.92 ± 0.37 | 0.75 ± 0.06 | 0.21 ± 0.09 |
| Brain | | 0.08 ± 0.01 | 0.08 ± 0.02 | 0.04 ± 0.02 | 0.01 ± 0.00 |
| Tumor | | 5.17 ± 1.48 | 5.36 ± 2.21 | 4.31 ± 0.30 | 2.69 ± 0.88 |

Organ distribution [^68^Ga] –PSMA CA030 at the time points: 10 min, 1 h, 4 h after injection. Values are expressed in % ID/g of tissue ± standard deviation; n = 3 for all tissues.


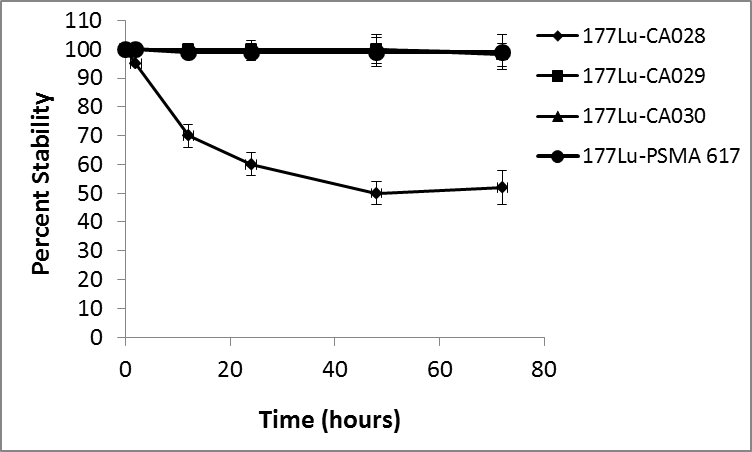


**Figure S3.** Serum stability of ^177^Lu-CA028, ^177^Lu-CA029, ^177^Lu-CA030 in comparison with ^177^Lu-PSMA-617 at 37 °C over 72 h (mean ± SD, n = 3) as determined by radio-ITLC.


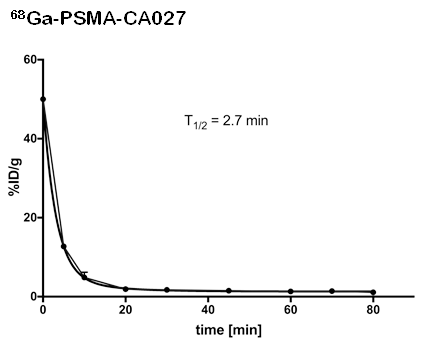

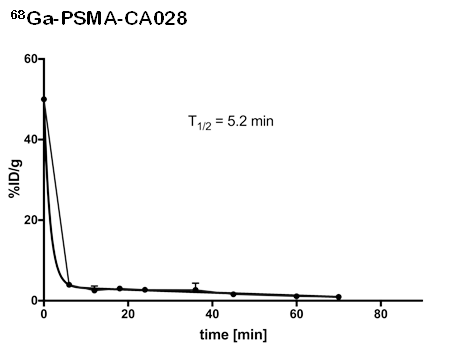


**Figure S4.** Blood time-activity curve for ^68^Ga PSMA-CA027 (0.6 nmol, 5 MBq) and ^68^Ga PSMA-CA028 (0.6 nmol, 6 MBq), including bi-exponential curve fit.

**Figure S5.** Organ distribution of 0.05 nmol of ^68^Ga-CA028 expressed as % ID/g of tissue ± SD (n = 3) at 20 min, 1 h, 2 h and 4 h after injection.

**Figure S6.** Intra-individual comparison of PSMA-PET performed either with ^18^F-PSMA-1007 (20 nmol, 218 MBq) 2 h p.i. (A) or ^68^Ga-CA028 (20 nmol, 339 MBq) 1 h and 3 h p.i. (B); maximum intensity projections, respectively.

**Figure S7.** Intra-individual comparison of ^68^Ga-CA030 PSMA-PET (A) with planar scans of ^177^Lu-PSMA-617 PSMA-therapy left anterior view (B), right posterior view (C).
